# At home and online during the early months of the COVID-19 pandemic and the relationship to alcohol consumption in a national sample of U.S. adults

**DOI:** 10.1371/journal.pone.0259947

**Published:** 2021-11-16

**Authors:** Karen G. Chartier, Jeanine P. D. Guidry, Catherine A. Lee, Thomas D. Buckley

**Affiliations:** 1 School of Social Work and Department of Psychiatry, Virginia Commonwealth University, Richmond, Virginia, United States of America; 2 Robertson School of Media and Culture, Virginia Commonwealth University, Richmond, Virginia, United States of America; 3 Schar School of Policy and Government, George Mason University, Fairfax, Virginia, United States of America; 4 School of Social Work, Virginia Commonwealth University, Richmond, Virginia, United States of America; National Cheng Kung University College of Medicine, TAIWAN

## Abstract

**Introduction:**

The current study aimed to understand the links between social media use and alcohol consumption during the early months of the COVID-19 pandemic.

**Method:**

Data were from the national Understanding American Study, a probability-based Internet panel weighted to represent the U.S. population. Subjects (N = 5874; 51% female) were adults, 18 years and older, who completed a March survey (wave 1) and a follow-up survey one month later (wave 3). Analyses assessed the relationships of social media use at wave 1 with wave 3 alcohol use frequency, accounting for wave 1 alcohol use frequency and the sociodemographic characteristics of the sample. Two alcohol use change variables were also assessed as outcomes–increased and decreased alcohol use between waves. We considered the effect of work status changes (working/studying from home and job loss) as potential moderators.

**Results:**

Twitter and Instagram users and users of multiple social media platforms, but not Facebook users, drank more frequently at wave 3. The results were similar when assessing relationships between social media use and increased alcohol use between waves. For Instagram users, more frequent alcohol use at wave 3 was at least partially attributed to drinking frequency at wave 1. Additionally, working/studying from home at wave 3 and employment (rather than job loss) were associated with greater consumption. The interaction effect between Twitter use and working/studying from home was statistically significant in association with alcohol use frequency at wave 3, as was the interaction effect between using multiple platforms and working/studying from home in association with decreased alcohol use between waves.

**Discussion:**

Exposure to content about COVID-19 and increased alcohol consumption during the pandemic may have contributed to more frequent alcohol use for some social media users. The study of public health messaging via social media to change alcohol use behaviors during traumatic events is warranted.

## Introduction

The U.S. population and other global regions began to see the economic, social, and psychological tolls, beyond the virus itself, months into the COVID-19 pandemic. There was a convergence of behaviors that presented some worrisome trends. In Australia, individuals who lost their jobs and who reported higher levels of stress, depression, sleep changes, and overeating had a greater chance of increased alcohol consumption [1]. A survey in Europe showed that more than 30% of participants changed their drinking behaviors during the early months of the COVID-19 pandemic, with some reporting lower and others higher alcohol consumption [2]. In the U.S., both increases and decreases in alcohol use were also observed, with increases being associated with COVID-19-related stress [3]. While there was an initial suggestion that challenged supply chains and store closures would result in a shortage of alcohol, many alcohol distributors and delivery services became creative to ensure that scarcity was not an issue [4]. In fact, there was a spike in wine and spirit sales as fears of COVID-19 grew [5]. There was even misinformation being spread that alcohol ingestion could help prevent or cure COVID-19, which was quickly discounted by the National Institutes of Health (NIH) and the World Health Organization (WHO) [6, 7].

Even prior to the COVID-19 pandemic, most U.S. adults used some type of social media [8]. Yet with the emergence of working from home and government and medical mandates on social distancing, home electronic usage, and more specifically social media usage, increased [9, 10]. Individuals who lost their job or shifted to remote work reported increased “screen time” use (e.g., television, computer, or smartphone) [11]. For some people, the increase in social media use was a way to stay up to date on COVID-19 and other news events [9]. Social media use was also described as a form of “escapism,” to alleviate stress and avoid difficult or problematic thoughts [12]. Additionally, social media use has positive healthcare implications by providing access to public health information and encouraging peer and emotional support [13], including for those who experience job loss to maintain their social networks [14].

However, the WHO [15] warned of an “infodemic” in the age of COVID-19, described as “an over-abundance of information–some accurate and some not–that makes it hard for people to find trustworthy sources and reliable guidance when they need it.” A study among a Chinese population showed that this increase in social media exposure during the COVID-19 outbreak correlated with an increase in anxiety and depression [16]. The use of social media as a source of information about the pandemic was also associated with using alcohol and other substances as a means to cope [17]. Alcohol-related content has long been prevalent on social media platforms [18], as evident by the trending hashtag #quarantini during the early days of the pandemic [19]. Prior studies suggest that both posting of and exposure to alcohol-focused content on social media is associated with higher rates of consumption and cravings, as well as clinical disorders [18, 20, 21].

Prior studies further show that traumatic events, stress, and alcohol consumption are interrelated. In a review article, Keyes et al. [22] reported that exposure to disastrous or traumatic events, whether manmade or natural, leads to an increase in overall alcohol consumption for those experiencing the stress, as well as serving as a coping mechanism for individuals with a history of alcohol use disorder. Wiederhold [9] suggested that in scenarios involving trauma or disaster, the number of fatalities will often be significantly outnumbered by the survivors who experience negative mental health outcomes as a result of the stress and trauma related to the disaster. Mechanisms found to be associated with increased alcohol use during COVID included financial difficulties, social isolation, and uncertainty about the future [23]. Specifically, external factors (e.g., loss of income, living alone) and internal factors (e.g., coping motives, pandemic related anxiety) were associated with drinking behaviors, including increased consumption [23, 24].

For the current study, we examined the relationship between alcohol consumption and social media use during the early months of the COVID-19 pandemic. As this review of relevant studies showed, there has been plentiful research on social media exposure and alcohol use and on traumatic events and alcohol use. What we know far less about is the relationship between social media use and alcohol consumption during a traumatic event. We further examined the effect of work status change, i.e., working/studying from home and unemployment during the COVID-19 pandemic as potential moderators of this relationship, given their association with increased social media exposure and COVID-19 related stressors. Concern was raised that the sudden isolation of millions and rise in unemployment would be associated with increased alcohol use [25]. Drawing from the reviewed areas of research and studies early on during the pandemic of social media exposure and mental health or substance use [16, 17], we expected that social media users would consume more alcohol, and that the positive relationship between social media use and alcohol consumption would be exacerbated for individuals who were working/studying from home or who lost their job. While most studies examining social media and alcohol use have been conducted in adolescents and college students or young adults [18], we extended this work by testing these relationships in an adult general population sample in the U.S.

## Methods

### Sample

The current sample (*N* = 5874) from the national Understanding American Study (UAS) included adults, 18 years and older. Begun in 2014, UAS is an ongoing, probability-based internet panel, with members recruited via address-based sample waves of all U.S. households, with internet-connected tablets provided to households not previously online [26]. A total of 6,933 panel members from the U.S.-weighted sample participated in a March 2020 survey, and then on April 1, 2020, were invited to participate in an ongoing coronavirus tracking survey. For the current study, we included those weighted sample members who completed both the wave 1 survey (March 10 and 31) and one month later the wave 3 survey (April 15 and 30). We did not include responses from the wave 2 survey, collected April 1 to 14, as we sought to examine alcohol use and change in alcohol use between these two time points early on in the COVID-19 pandemic. Surveys were completed online using a computer, mobile phone, or tablet and in English or Spanish according to the respondent’s preference. The participation rate was 82% in March for the weighted sample, and approximately 96% of respondents completed their wave 3 survey in April [26, 27]. Data are weighted to account for the sampling procedure and probabilities of selection and to be representative of the U.S. population, as described elsewhere in more detail [26, 27].

### Measures

**Social media use** was collected at wave 1. Our cross-platform approach to studying social medial use focused on two dimensions, i.e., the effects of using individual platforms and of using multiple platforms, and the association with alcohol use [28]. Respondents indicated whether they used Twitter, Instagram, and/or Facebook, with each item coded 0 as no and 1 as yes. Affirmative responses indicated that respondents had an account and used it, while those indicating that they had an account, but never used it, and those who did not have an account were coded no. A combined social media use variable identified respondents who used multiple platforms (2 or 3 platforms; coded 1) compared to those who did not use social media or only 1 platform (coded 0). Additionally, active users reported their average minutes on social media per day across platforms. Because responses were not capped in the survey to the total number of minutes in a day, responses over 1440 minutes were re-coded to 1440 to indicate near constant social media use. Respondents reporting no social media use had zero minutes. To address the positive skew of the reported minutes of social media use, we created a low to high quartiles of social media use variable to include in regression analyses.

**Alcohol use frequency**, collected at wave 1 and wave 3, measured the number of days in the past seven days (0 to 7) that respondents consumed alcohol. As other studies showed both increases and decreases in alcohol use attributed to the COVID-19 pandemic [2, 3], we also constructed two binary alcohol use variables to identify those respondents (1) with increased and (2) with decreased frequency of use. These variables were created by subtracting the wave 3 number of days used from the wave 1 number of days used. Respondents with values above zero were categorized as having increased use (0 = no and 1 = yes) and with values less than zero as having decreased use (0 = no and 1 = yes).

#### Work status

Respondents were asked at wave 1 and wave 3 to indicate if they worked or studied from home in the last seven days to keep safe from the coronavirus, with response options being 0 = no and 1 = yes. Respondents also indicated whether they were currently employed at both waves. We combined their responses into the following three employment categories to indicate whether they: 1 = lost their job between wave 1 and wave 3; 2 = did not have a job at both waves; or 3 = were either employed at both waves or gained employment between wave 1 and wave 3.

**Sociodemographic variables** at wave 1 included sex (male or female), race/ethnicity, marital status (married, separated/divorced/widowed, or never married), level of education (less than high school, high school/GED, some college, and a bachelor’s degree or higher), age categorized into groups (18–29, 30–44, 45–64, and 65 years or more), and the number of members in each respondent’s household. The race/ethnicity variable combines respondents’ answers to questions about race and about Hispanic/Latin ethnicity into five groups (non-Hispanic Asian, non-Hispanic Black, Hispanic/Latino, non-Hispanic White, and non-Hispanic individuals of other races).

### Data analysis

Analyses were conducted using Stata’s SVY command [29] to account for the UAS survey’s weight information. Descriptive statistics on all variables were generated for the study sample. Bivariate tests, *t* tests for continuous variables and chi-square for categorical variables, compared social media users on sociodemographic characteristics at wave 1 and on alcohol consumption and work status variables at wave 1 and wave 3. These tests looked at Twitter, Instagram, and Facebook use separately as well as respondents’ use of multiple social media platforms.

Multivariate models examined three outcomes: (1) alcohol use frequency at wave 3 and (2) increased and (3) decreased alcohol use between waves. Hierarchical linear regression was used when examining the first outcome. Models were tested in three steps: first (model 1) to examine social media use and work status indicators as independent variables; then (model 2) to re-examine these variables after accounting for respondents’ alcohol consumption at wave 1; and, next (model 3), after accounting for sociodemographic variables. We included these control variables in models 2 and 3 to rule out other possible explanations; it is possible that wave 3 drinking behaviors were a continuation of wave 1 drinking behaviors [30] and that the association between social media use and drinking outcomes reflected differences in the sociodemographic characteristics of social media users and non-users and between different social media platforms [8]. We controlled for the selected socio-demographics after preliminary analyses (see S1 Table) showed that respondents who did and did not use Twitter, Instagram, and Facebook or did and did not use multiple platforms were different on these variables.

Next, we used hierarchical logistic regression to examine the two alcohol use change variables (increased drinking and decreased drinking). These models did not control for alcohol use frequency at wave 1, which we used in combination with the wave 3 alcohol frequency measure to create both of these outcome variables. Model 1 examined social media use and work status indicators as independent variables; and model 2 to re-examined these variables after accounting for sociodemographic characteristics.

In follow up analyses, in recognition that many people use more than one social media platform, we repeated these regression analyses replacing the three separate platform variables with a combined variable comparing respondents who used multiple platforms with those who did not. Lastly, we tested interactions between social media use and the working/studying from home variable at wave 3. We added one interaction term at a time for Twitter, Instagram, and Facebook use and for the use of multiple platforms. We did not test interaction effects with working/studying from home at wave 1 or job loss after these variables were not consistently associated with alcohol consumption in main effect models. We plotted statistically significant interaction effects. A probability value of .05 was used to determine statistical significance. All reported coefficients are unstandardized.

## Results

### Sample characteristics at wave 1

Table 1 presents the sociodemographic characteristics of the sample. About half of respondents were female, and the majority were non-Hispanic White, married, 40 years of age or older, and had at least some college education. The average number of household members was slightly under two. To consider how the study sample was similar to the U.S. population, we compared these statistics against 2019 QuickFacts (the most recent year publicly available) from the U.S. Census Bureau [31]. As such, our sample characteristics were comparable with 2019 census estimates in regards to sex, but included a somewhat larger percentage than in the U.S. population of non-Hispanic Whites, persons with a bachelor’s degree, and those 65 years and older, respectively, 60.1%, 31.5%, and 16.5% in the U.S. population [31]. The number of individuals per household was somewhat smaller in the current sample than the 2019 U.S. population estimate, which was 2.63 [31].

**Table 1:** Sample socio-demographics at wave 1 (*N* = 5874).

	% or *M* (*SE*)
**Sex (% Female)**	50.91
**Age (%)**	
18–29 years	12.20
30–44 years	32.11
45–64 years	34.86
65 years or older	20.82
**Race/ethnicity (%)**	
Asian^+^	5.25
Black^+^	11.53
Hispanic/ Latino	15.35
White^+^	64.44
Other groups^+^	3.43
**Education (%)**	
Less than high school	8.45
High school/ GED	28.93
Some college	27.96
Bachelor’s degree or more	34.66
**Marital status (%)**	
Married	56.72
Previously married^++^	19.26
Never married	24.01
**Number of household members, *M* (*SE*)**	1.78 (.028)

*Notes*: *M* = mean; *SE* = standard error; GED = general equivalency diploma;

^+^non-Hispanic;

^++^separated/divorced/widowed

### Social media use at wave 1

Most respondents in the sample used Facebook (67.98%), with fewer using Instagram (32.50%) or Twitter (19.61%). On average, respondents spent one hour per day on social media (*M* = 61.04, *SE* = 1.82), although this was higher for Twitter users (*M* = 100.69, *SE* = 6.31) and Instagram users (*M* = 97.79, *SE* = 4.23) than for Facebook users (*M* = 80.02, *SE* = 2.43). Thirty-three percent of respondents in the current sample reported using more than one social media platform (0 platforms 26.39%; one 39.97%; two 20.85%; and three 12.80%).

### Alcohol use and work status at waves 1 and 3 by social media use

Table 2 presents the relationships between social media use at wave 1 and alcohol use frequency at both wave 1 and 3 and change in alcohol use (increased or decreased) between waves. Relationships of social media use and work status indicators (working/studying from home at waves 1 and 3 and change in employment status between waves) are also shown. Respondents reported drinking, on average, one day over the past 7 days at wave 1 and somewhat more than one day at wave 3. Additionally, a greater percentage of respondents showed increased alcohol use than decreased alcohol use between waves. Alcohol consumption did not vary by social media use at wave 1, but was significantly greater at wave 3 for Twitter users and Instagram users compared to non-users. Alcohol use frequency was also significantly greater at wave 3 for respondents who used 2 or 3 social media platforms than for those using fewer. A significantly larger percentage of Twitter users and users of multiple platforms also increased their drinking between waves, while a larger percentage of Instagram users than non-users both increased and decreased their alcohol use between waves. There were no statistically significant differences for the alcohol use outcomes between Facebook users and non-users.

**Table 2:** Alcohol use and work status by social media use (*N* = 5874).

	Total	Twitter User		Instagram User		Facebook User		More than One Platform	
		*No*	*Yes*	*No*	*Yes*	*No*	*Yes*	*No*	*Yes*
**Days consumed alcohol in past week**									
Wave 1, *M* (*SE*)	1.19 (.034)	1.17 (.038)	1.26 (.072)	1.15 (.041)	1.27 (.059)	1.25 (.062)	1.16 (.040)	1.16 (.042)	1.25 (.057)
Wave 3, *M* (*SE*)	1.49 (.037)	1.44 (.041)	1.70 (.086)**	1.43 (.045)	1.62 (.067)*	1.60 (.070)	1.44 (.044)	1.43 (.045)	1.62 (.065)*
**Alcohol use change** ^++^									
Increased (% Yes)	26.67	25.03	33.41***	24.16	31.86***	25.84	27.05	24.03	31.81***
Decreased (% Yes)	12.16	12.02	12.70	11.14	14.25*	10.73	12.82	11.23	13.98
**Working / studying from home**									
Wave 1 (% Yes)	26.54	22.44	35.09***	23.09	33.64***	25.84	26.88	23.10	33.29***
Wave 3 (% Yes)	51.04	47.88	63.97***	46.65	60.14***	47.86	52.60*	46.47	60.16***
**Employment change** ^++^									
No longer employed (%)	12.11	12.40	10.95***	11.65	13.07***	11.27	12.52***	11.70	12.96***
Not employed at both waves (%)	38.04	40.57	27.66	43.41	26.89	45.74	34.38	43.60	26.98
Employed (%)	49.85	47.04	61.39	44.94	60.04	42.99	53.10	44.70	60.06

*Notes*: *M* = mean; *SE* = standard error;

^++^Change from wave 1 to wave 3;

*p < .01;

**p < .01;

***p < .001

Related to work status, about a quarter of all respondents were either working or studying from home at wave 1, and this increased to about half of respondents at wave 3. At both wave 1 and wave 3, a significantly larger percentage of Twitter and Instagram users compared to non-users and a larger percentage of multiple platform users compared those using fewer were working/studying from home. For Facebook users compared to non-users, this was only the case at wave 3. Additionally, most respondents did not have a change in their employment status, either being employed or not reporting a current job at both waves. About 12% of respondents changed their employment status from having a current job at wave 1 to not having a job at wave 3. The relationships between social media use and employment change were statistically significant across all social media variables. Such that, a greater percentage of social media users were employed and a smaller percentage of social media users did not have a job at both waves, while the percentages for those who lost their job were similar between social media users and non-users.

### Multivariate models

#### Linear regression: Alcohol use frequency at wave 3

See Table 3, model 1; we first regressed alcohol use frequency at wave 3 on social media use at wave 1 and the two work status variables. Instagram use and time spent on social media across platforms were statistically significant, but in opposite ways. Being an Instagram user was associated with more frequent drinking, while more time spent on social media across platforms was associated with less frequent drinking. Working/studying from home at wave 3, but not wave 1, was associated with consuming alcohol more frequently at wave 3. Conversely, respondents who were no longer employed at wave 3 or not employed at both waves consumed alcohol less frequently than respondents who were employed.

**Table 3:** Hierarchical linear regression models for wave 3 alcohol use frequency.

	Model 1 ^a^	Model 2 ^b^	Model 3 ^c^
	*B* (*SE*) [95% CI]	*B* (*SE*) [95% CI]	*B* (*SE*) [95% CI]
**Social media use**			
Twitter user (ref: No)	.184 (.104) [-.019, .338]	.148 (.072) [.008, .289]*	.081 (.071) [-.059, .221]
Instagram user (ref: No)	.241 (.091) [.062, .421]**	.081 (.062) [-.042, .203]	.139 (.064) [.013, .265]*
Facebook user (ref: No)	-.100 (.094) [-.284, .085]	-.085 (.059) [-.200, .030]	-.129 (.059) [-.244, -.014]*
Quartiles of minutes spent on social media	-.147 (.039) [-.224, .070]***	-.034 (.026) [-.085, .017]	-.005 (.026) [-.056, .045]
**Work status**			
Working/ studying from home—wave 1 (ref: No)	-.102 (.093) [-.284, .080]	-.098 (.065) [-.226, .029]	-.080 (.066) [-.210, .048]
Working/ studying from home—wave 3 (ref: No)	.339 (.079) [.184, .495]***	.215 (.055) [.107, .324]***	.181 (.056) [.072, .291]**
Employment change (ref: Employed)			
No longer employed	-.237 (.119) [-.471, -.003]*	-.082 (.085) [-.249, .086]	-.030 (.085) [-.197, .136]
Not employed at both waves	-.177 (.080) [-.333, -.021]*	-.113 (.053) [-.217, -.009]*	-.064 (.059) [-.180, .053]
**Wave 1 alcohol use**			
Days consumed alcohol, past week	––	.837 (.017) [.804, .870]***	.824 (.018) [.790, .859]***
**Sociodemographic variables**			
Female (ref: Male)	––	––	-.094 (.056) [-.206, .016]
Age (ref: 65 +)			
18–29	––	––	-.010 (.111) [-.228, .208]
30–44	––	––	.046 (.085) [-.120, .212]
45–64	––	––	.075 (.067) [-.055, .245]
Race/ethnicity (ref: White^+^)			
Asian^+^	––	––	-.501 (.110) [-.716, -285]***
Black^+^	––	––	-.102 (.105) [-.308, .104]
Hispanic/ Latino	––	––	-.069 (.086) [-.238, .100]
Other^+^	––	––	-.181 (.118) [-.411, .050]
Education (ref: Bachelor’s degree or more)			
< High school	––	––	-.194 (.102) [-.394, .005]
High school/ GED	––	––	-.257 (.074) [-.401, -.112]***
Some college	––	––	-.189 (.068) [-.322, -.057]**
Marital status (ref: Never married)			
Married	––	––	.161 (.076) [.013, .309]*
Previously married^++^	––	––	.156 (.092) [-.039, .042]
Number of household members	––	––	.001 (.021) [.013, .309]

*Notes*: GED = general equivalency diploma;

^+^non-Hispanic;

^++^Separated/divorced/widowed;

^a^Model includes social media and work status variables;

^b^Model 1 + alcohol use frequency at wave 1;

^c^Model 2 + sociodemographic variables; and

*p < .05;

**p < .01;

***p < .001.

For model 2, we re-evaluated these relationships after accounting for wave 1 alcohol consumption, which was significant and positively associated with later alcohol use frequency at wave 3. Instagram use and time spent on social media across platforms were no longer associated with alcohol use at wave 3, while Twitter use was now associated with drinking more frequently. The variance explained by working/studying from home at wave 3 was reduced, but remained statistically significant and associated with greater alcohol use frequency. Respondents who did not have a current job at both waves again drank less frequently than those who were employed. The difference in drinking frequency between respondents who lost their job and who were employed was no longer statistically significant.

Some of these relationships again changed after including sociodemographic variables in model 3. Whites, respondents with a four-year college degree or more, and those who were married drank more frequently than, respectively, Asians, those with a high school education or some college, and being never married. Wave 1 alcohol use frequency continued to predict an increased frequency of alcohol at the later wave with relatively no change in the variance explained. Instagram use was again positively associated with alcohol use frequency at wave 3, although the variance explained was reduced. Facebook use was now associated with less alcohol consumption, and Twitter use was no longer associated. Again, the variance explained by working/studying from home at wave 3 was reduced, but it remained statistically significant and positively associated with alcohol consumption. For employment status, respondents who lost their job at wave 3 or were not working at both waves were not significantly different in terms of their frequency of alcohol use than respondents who were employed.

#### Logistic regression: Increased and decreased alcohol use

Table 4 shows the results of the hierarchical logistic regression models for the two alcohol use change variables. First, for increased alcohol use, in model 1, Instagram use and working/studying from home at wave 3 were statistically significant and positively associated. Conversely, when compared to being employed, not having a current job at both waves was statistically significant and negatively associated with increased alcohol use. The relationship between job loss at wave 3 and increased alcohol use was not statistically significant, and neither were the other social media use variables nor working/studying from home at wave 1. Additionally, these relationships did not change when controlling for sociodemographic characteristics in model 2, which showed that Whites compared to Asians, having a college education or more compared to less education, and being married compared to never married were significantly and positively associated with increased alcohol use.

**Table 4:** Hierarchical logistic regression models for alcohol use change variables.

	Increased alcohol use		Decreased alcohol use	
	Model 1 ^a^	Model 2 ^b^	Model 1 ^a^	Model 2 ^b^
	*B* (*SE*)	*B* (*SE*)	*B* (*SE*)	*B* (*SE*)
	[95% CI]	[95% CI]	[95% CI]	[95% CI]
**Social media use**				
Twitter user (ref: No)	.201 (.110)	.123 (.113)	-.133 (.153)	-.194 (.159)
	[-.016, .417]	[-.098, .344]	[-.432, .167]	[-.505, .117]
Instagram user (ref: No)	.270 (.100)	.314 (.106)	.222 (.133)	.244 (.144)
	[.073, .467]**	[.107, .521]**	[-.039, .483]	[-.038, .526]
Facebook user (ref: No)	-.102 (.103)	-.150 (.107)	.048 (.142)	.127 (.144)
	[-.305, .101]	[-.360, .060]	[-.230, .326]	[-.155, .410]
Quartiles of minutes spent on social media	.006 (.044)	.045 (.047)	.062 (.064)	.078 (.067)
	[-.081, .093]	[-.046, .136]	[-.064, .188]	[-.053, .208]
**Work status**				
Working/ studying from home—wave 1 (ref: No)	-.182 (.101)	-.153 (.103)	.152 (.134)	.117 (.136)
	[-.379, .016]	[-.356, .049]	[-.110, .414]	[-.151, .384]
Working/ studying from home—wave 3 (ref: No)	.404 (.090)	.356 (.095)	-.072 (.123)	-.056 (.128)
	[.228, .580]***	[.170, .541]***	[-.312, .168]	[-.308, .195]
Employment change (ref: Employed)				
No longer employed	-.072 (.130)	.024 (.135)	.060 (.183)	.096 (.183)
	[-.326, .182]	[-.240, .290]	[-.299, .419]	[-.263, .455]
Not employed at both waves	-.343 (.092)	-.217 (.106)	-.147 (.126)	-.015 (.149)
	[-.523, -.164]***	[-.424, -.010]*	[-.393, .099]	[-.307, .276]
**Sociodemographic variables**				
Female (ref: Male)	––	-.117 (.090)	––	-.336 (.123)
		[-.293, .059]		[-.577, -.095]**
Age (ref: 65 +)				
18–29	––	.252 (.195)	––	-.201 (.274)
		[-.129, .634]		[-.739, .337]
30–44	––	.246 (.146)	––	-.008 (.201)
		[-.040, .532]		[-.402, .386]
45–64	––	.232 (.123)	––	.093 (.168)
		[-.010, .473]		[-.423, .237]
Race/ethnicity (ref: White^+^)				
Asian^+^	––	-.643 (.241)	––	.217 (.266)
		[-1.116, -.170]**		[-.305, .739]
Black^+^	––	-.026 (.157)	––	.165 (.193)
		[-.334, .283]		[-.214, .543]
Hispanic/ Latino	––	-.099 (.144)	––	-.191 (.201)
		[-.381, .183]		[-.585, .204]
Other^+^	––	-.311 (.237)	––	-.282 (.347)
		[-.775, .152]		[-.961, .398]
Education (ref: Bachelor’s degree or more)				
< High school	––	-.438 (.213)	––	-.518 (.299)
		[-.856, -.020]*		[-1.104, .068]
High school/ GED	––	-.314 (.119)	––	-.073 (.161)
		[-.547, -.080]**		[-.388, .242]
Some college	––	-.183 (.102)	––	-.161 (.146)
		[-.382, .017]**		[-.447, .124]
Marital status (ref: Never married)				
Married	––	.332 (.127)	––	-.708 (.170)
		[.084, .581]**		[-1.040, -.376]***
Previously married^++^	––	.194 (.149)	––	-.592 (.198)
		[-.100, .487]		[-.979, -.205]**
Number of household members	––	-.026 (.034)	––	.014 (.046)
		[-.092, .040]		[-.077, .105]

*Notes*: GED = general equivalency diploma;

^+^non-Hispanic;

^++^Separated/divorced/widowed;

^a^Model includes social media and work status variables;

^b^Model 1 + sociodemographic variables; and

*p < .05;

**p < .01;

***p < .001.

For decreased alcohol use, none of the social media use and work status variables were significantly associated in model 1 or model 2. Sociodemographic variables significantly associated with decreased alcohol use were sex and marital status. Being male and never married, respectively, compared to females and being married or previously married were positively associated with decreased use.

#### Follow-up analyses

*Regression models*: *Use of multiple social media platforms*. We re-analyzed the above regressions models using a combined social media variable comparing two groups, respondents who used multiple platforms and respondents who used 1 platform or no social media. The full results of these models are not shown; we instead focus on reporting on the use of multiple social media platforms in association with alcohol use. First, in associate with alcohol use frequency at wave 3, model 1 (including the social media and work status variables) showed that using multiple social media platforms at wave 1 was statistically significantly associated with more frequent alcohol use (*B* = .285, *SE* = .085 [95% CI .118, .452] and *p* = 0.001). This relationship remained statistically significant after controlling for wave 1 drinking frequency (*B* = .131, *SE* = .060 [95% CI .014, .249] and *p* = 0.028) and was a trend effect (*p* < .10) after controlling for sociodemographic characteristics (*B* = .117, *SE* = .060 [95% CI < -.001, .234] and *p* = 0.051).

For increased alcohol use, in model 1 (including the social media and work status variables), using multiple social media platforms at wave 1 was statistically significant and positively associated (*B* = .323, *SE* = .093 [95% CI .141, .505] and *p* = 0.001), and this relationship remained statistically significant in model 2 after controlling for sociodemographic characteristics (*B* = .270, SE = .097 [95% CI .080, .460] and *p* = 0.005). Conversely, the use of multiple social media platforms was not associated with decreased alcohol use in model 1 (*B* = .153, *SE* = .131 [95% CI -.105, .410] and *p* = 0.246) or model 2 (*B* = .058, *SE* = .154 [95% CI -.244, .360] and *p* = 0.706).

**Fig 1:**
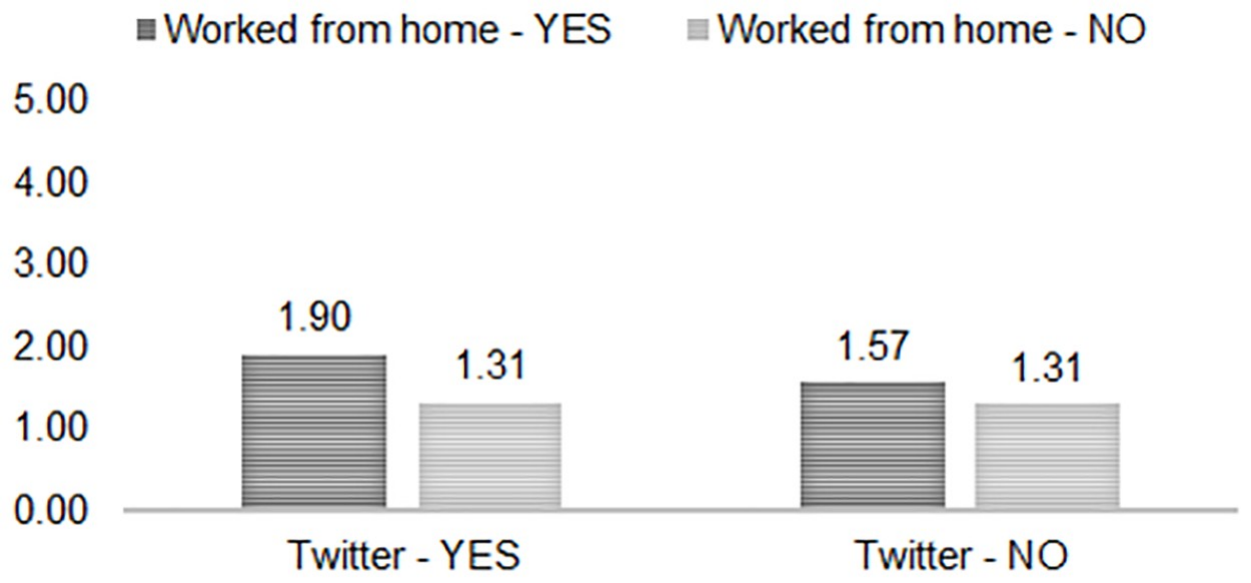
Twitter use by working/studying from home: Mean days drinking in past week.

*Moderating effect of working/studying from home at wave 3*. When testing whether working/studying from home at wave 3 moderated the relationships between social media use and alcohol consumption (models not shown), the interaction between Twitter use and working/studying from home was statistically significant (*B* = .270, *SE* = .132 [95% CI .011, .530] and *p* = 0.041) in association with wave 3 alcohol use frequency. The interaction effects including Instagram use (*B* = .167, *SE* = .114 [95% CI -.057, .395] and *p* = 0.144), Facebook use (*B* = .015, *SE* = .107 [95% CI -.190, .225] and *p* = 0.887) and the use of multiple social media platforms (*B* = .160, *SE* = .111 [95% CI -.058, .378] and *p* = 0.151) with working/studying from home at wave 3 were not significantly associated. Fig 1 provides a visual representation of the Twitter use by working/studying from home interaction. Respondents working/studying from home at wave 3 compared to those who were not consumed alcohol more frequently at wave 3, and this relationship was strongest for those who used Twitter.

**Fig 2:**
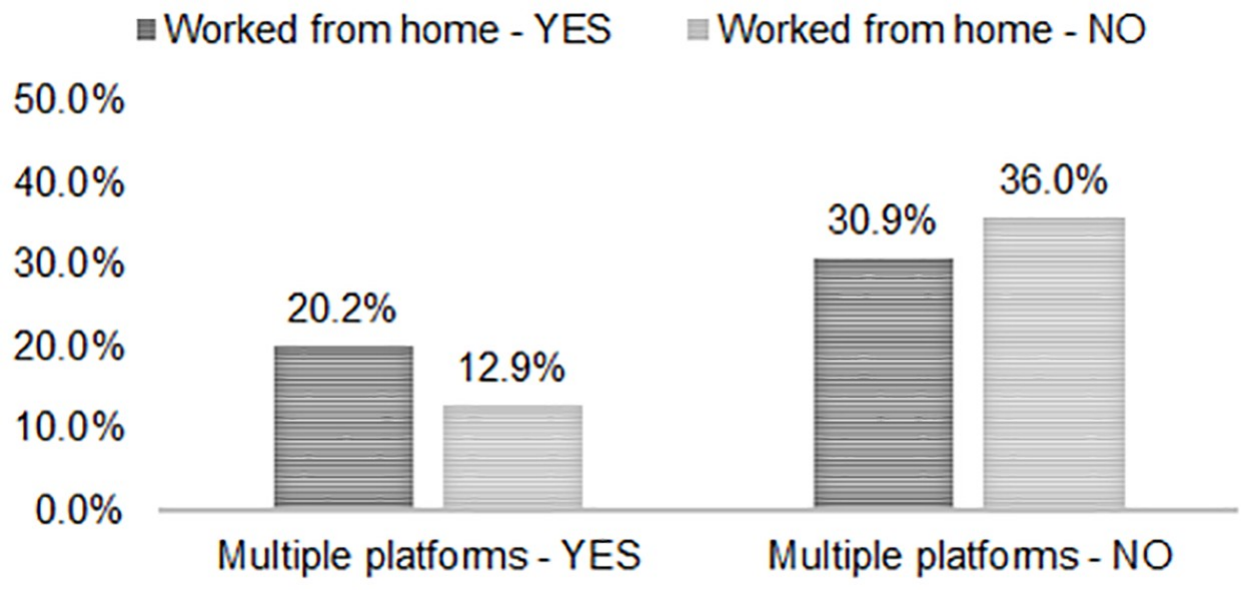
Multiple platforms use by working from home: Decreased alcohol use (%).

Conversely, social media by working/studying at home interaction effects were not statistically significant in models for increased alcohol use: Twitter use (*B* = .234, *SE* = .221 [95% CI -.200, .667] and *p* = 0.291); Instagram use (*B* = .003, *SE* = .179 [95% CI -.348, .354] and *p* = 0.988); Facebook use (*B* = .017, *SE* = .186 [95% CI -.347, .381] and *p* = 0.927); and multiple platforms use (*B* = .086, *SE* = .1781 [95% CI -.234, .437] and *p* = 0.629). While for decreased alcohol use, the multiple platforms use by working/studying from home interaction effect was statistically significant (*B* = -.619, *SE* = .257 [95% CI -1.123, -.115] and *p* = 0.016), but not the interaction effects with individual platforms, i.e., Twitter use (*B* = -.296, *SE* = .292 [95% CI -.868, .275] and *p* = 0.309), Instagram use (*B* = -.319, *SE* = .243 [95% CI -.795, .157] and *p* = 0.180), and Facebook use (*B* = -.067, *SE* = .253 [95% CI -.563, .428] and *p* = 0.790). Fig 2 provides a visual representation of the multiple platforms use by working/studying from home at wave 3 interaction. In this case, a smaller percentage of respondents using multiple social media platforms showed decreased alcohol use compared to those who did not use multiple platforms, and this relationship was strongest for those who were *not* working/studying from home.

## Discussion

We assessed in a U.S. general population sample of adults how social media use was associated with alcohol consumption during the months of March and April 2020 (UAS survey waves 1 and 3), when many individuals were sheltering at home because of the COVID-19 pandemic. The current study focused on two aspects of social media use at wave 1 [28], comparing the use of both individual platforms (Instagram, Twitter, and/or Facebook) and the use of multiple platforms, and examined three alcohol use outcomes–alcohol use frequency at wave 3 and increased and decreased alcohol use between waves. With earlier studies showing increased drinking in association with traumatic events [22] and with greater social media exposure [18, 20], we expected that social media use would be associated with greater alcohol use in this sample. The hypothesis was supported, but not for all social media platforms. At wave 3, Twitter and Instagram users drank alcohol more frequently than non-users. There was no difference in how frequently Facebook users and non-users drank alcohol in bivariate tests, and in multivariate models Facebook users drank less. These results to a great extent were replicated when social media variables were examined in association with increased alcohol use between waves, and with users of multiple platforms, who drank more frequently than users of only one platform or none. Social media use was not associated with decreased alcohol use in main effects models. As reported by other studies conducted in the early stages of the COVID-19 pandemic [2, 3], we observed both increased and decreased alcohol consumption. However, a greater percentage of respondents increased their drinking (27%) than decreased their drinking (12%).

Two potential mechanisms for these diverging effects, related to increased distress and decreased availability of alcohol, were discussed by Rehm et al. [23] in a commentary on anticipated alcohol use during the pandemic. It could be that Twitter and Instagram users were exposed to more social media content about the COVID-19 pandemic, which Gao et al. [16] found in China was associated with a high prevalence of mental health problems. A 2017 meta-analysis [32] of experimental studies provides support for this mechanism, similarly showing a link between media exposure on disasters and other large-scale traumatic events, including via both mainstream sources and the internet, and negative psychological outcomes. Similarly, in the U.S. and Canada, using social media to stay informed about the COVID-19 pandemic was associated with using alcohol and other substances to cope with pandemic-related stress [17]. Findings of compulsive social media use associated with COVID-19-related distress also emerged in studies conducted in Europe [33, 34].

Additionally, media reports that alcohol consumption greatly increased in conjunction with stay at home orders were widely disseminated (e.g., Fulmer [19]), and, thus, individuals using Twitter and Instagram may have had greater exposure to these stories and to alcohol-related content on social media. The positive relationship between using multiple social media platforms and consuming more alcohol may further indicate that some respondents had greater exposure to this content. There is an extensive body of research showing that drinking behaviors are positively influenced by descriptive norms [35], which are individuals’ perception of how much other people are drinking, and which are often overestimated [36]. At least in adolescents, higher descriptive alcohol use norms mediate the positive relationship between exposure to substance-related social media content and alcohol use [37]. With few exceptions (e.g., Lee et al. [38]), however, these relationships have not been widely examined among individuals at developmental stages beyond adolescence and young adulthood.

Additionally, while the use of multiple social media platforms was robustly associated with consuming more alcohol, the relationship between alcohol use and the use of individual platforms changed depending on other variables included in the model. The increased frequency of alcohol consumption for Instagram users at wave 3 was at least partially attributed to differences in wave 1 drinking and, for Twitter users, partially attributed to their sociodemographic characteristics, as the effects of Instagram use and Twitter use were no longer statistically significant when these variables were added to the model. In particular, Instagram users are on average among the youngest users on social media platforms, while Facebook use is common across a range of ages and Twitter use is more common in middle-aged adults [8, 39]. The prevalence of risky alcohol use also trends younger, with rates of binge drinking peaking in young adulthood and decreasing thereafter [40]. For demographic effects, those respondents with a bachelor’s degree or higher drank more frequently at wave 3 than those with lower levels of education. Twitter users in supplemental analyses showed the highest percentage of individuals with a bachelor’s degree or higher, which may contribute to their increased drinking at wave 3.

It was unexpected that respondents who reported more time on social media at wave 1 drank alcohol less frequently at wave 3, although this relationship appears to also be partially attributed to how often they were drinking at wave 1. Given the lower percentage of respondents working/studying from home at wave 1 (27%-35%) compared to wave 3 (53%-64%), it could be that those individuals who were drinking more frequently at wave 1 were less likely to be at home and on social media. Time on social media at wave 1 was not associated with increased or decreased drinking between waves. Additionally, it was unexpected that job loss was not associated with greater alcohol use, given prior studies linking alcohol use during the pandemic to employment-related changes [24]. In fact, we found the opposite; such that, being employed was associated with both drinking more frequently at wave 3 and increased drinking between waves, and not with decreased drinking between waves.

For the current study, we further hypothesized that the positive relationship between social media use and alcohol consumption would be stronger for individuals who experienced a change in their work status, and we tested the moderating effect of working/studying from home. This hypothesis was supported for Twitter users, but not for Instagram, Facebook, or multiple platforms users. For Twitter use, the combination with working/studying from home was associated with more frequent drinking at wave 3 but not with increased alcohol use between waves. In our sample, a larger percentage of Twitter users, Instagram users, and multiple platforms users compared to non-users were working/studying from home at waves 1 and 3, and a somewhat larger percentage of Facebook users compared to non-users were working/studying from home at wave 3. It could be that this interaction effect was not shown for Instagram users and multiple platforms users because most of them skew younger, and young Millennials and older members of Gen Z that tend to use Instagram more were increasingly moving home with their parents because of the COVID-19 related economic downturn, where they may have had less access to alcohol [41, 42]. Rehm et al. [23] pointed to this reduced access as being one potential mechanism influencing alcohol use during the pandemic, which, suggested by this study, may be more likely to affect younger adults than middle-aged adults. This mechanism could further explain the interaction observed between using multiple platforms and working/studying from home for decreased alcohol use. For example, White et al. [41] showed that while college students who moved home drank less, those who continued to live with peers significantly increased their drinking. Similarly, in the current study, the *smallest* percentage of respondents to decrease their alcohol use between waves were those using multiple social media platforms and not working/studying from home, where they expectedly had more access to alcohol than their homebound peers.

### Strengths and limitations

The current study has some unique strengths. Because of the timeliness of the UAS data collections, we were able to use current data on social media use and alcohol consumption during the early months of the COVID-19 pandemic, the depth and breadth of which was not readily available via other sources. Self-reported alcohol use and work status variables were available across two time points, which allowed for an analysis of changes in behavior and work situations. The longitudinal nature of the data also enabled the establishment of time order between social media use at wave 1 and alcohol use at wave 3. The analysis was completed in a weighted sample, making the results generalizable to the U.S. population; however, the focus on older groups and individuals more likely to be engaged in post-secondary education may make the results less generalizable to more vulnerable groups and those under 18 years of age, for whom social media use and alcohol consumption habits and impacts may differ.

This study is not without limitations. Our secondary analysis of UAS data used variables to examine relationships between social media use, alcohol consumption, and work status changes not specifically designed for this purpose. Future studies using measures with strong psychometric properties are needed to confirm our findings. The UAS gathered information on the frequency of alcohol use at both waves but not the amount consumed, which is typical of epidemiologic studies that cover a range of topics [43]. As a result, it was clear how many days per week respondents consumed alcohol, but not the overall quantity of consumption. And while higher alcohol use frequency may be indicative of more problematic use, other related measures (quantity and alcohol use-related problems) would help to identify individuals with greater risk of adverse health outcomes [43]. Additionally, social media use was only measured at wave 1 and was also not categorized by function, so different populations may have used social media for different purposes, the content of which (e.g., whether alcohol-related or COVID-19-related) was not included in the study. We also do not know whether respondents’ social media use changed between waves, in a way that was similar to other studies conducted in this timeframe showing increased social media use [9–11, 33, 34].

### Implications for prevention

Regardless of the cause of increased alcohol use, even a moderate increase can have economic, health, and social implications. In the U.S., alcohol use and misuse have been attributed to significant economic losses. Sacks et al. [44] found that in 2010, alcohol use cost the U.S. approximately $249.0 billion; 72% of the costs were associated with reduced workplace productivity and 11% to healthcare expenses. Alcohol consumption has also been linked to negative short- and long-term health effects and effects on mortality measures, ranging from increases in alcohol-related cancers to alcohol-related accidents [45].

Most relevant to the pandemic and COVID-19 is scientific evidence that alcohol use reduces immune functioning [46], making individuals who drank more heavily more susceptible to infection. As such, the current study has implications for prevention strategies regarding COVID-19 and alcohol consumption. Social media platforms are increasingly being used for disease detection and outbreak tracking allowing epidemiologists and public health researchers rapid detection of infectious diseases and their spread [47–49], as well as for identifying at-risk drinkers and for the delivery by web-based interventions [50]. For these reasons, measurements of alcohol use and social media use, as well as their correlation, may be relevant in the prevention, tracking, and treatment.

During the pandemic, and for some a concurrent “infodemic,” social media could be used as a resource to provide access to otherwise unavailable public health information, as well as peer and emotional support around health and safety. In studying social media’s use in health communication, Moorhead et al. [13] found that social media improved the tailoring, sharing, and availability of health communication. However, it was also found that much health information shared via social media lacked reliability and quality that may deter vulnerable populations from seeking care [13]. Given this information, the potential reach and impact of public health messaging via social media regarding alcohol consumption should not be understated, and the application of social media campaigns (e.g., Perkins et al. [51]) that seek to influence alcohol use behaviors warrants future research under traumatic conditions.

## Supporting information

S1 Table Sample socio-demographics by Twitter, Instagram, Facebook, and multiple social media platform use (*N* = 5874). (DOCX)
