# Supplementary material for: At home and online during the early months of the COVID-19 pandemic and the relationship to alcohol consumption in a national sample of U.S. adults
Source: PLoS One. 2021 Nov 16;16(11):e0259947. doi: 10.1371/journal.pone.0259947 (PMC8594812; doi:10.1371/journal.pone.0259947)
Supplement: S1 Table — (DOCX) [file pone.0259947.s001.docx]

**Table S1.** **Sample Socio-demographics by Twitter, Instagram, Facebook, and multiple social media platform use (*N*=5874)**

|  | **Did not use Twitter** | **Used**  **Twitter** | ***p*** | **Did not use Instagram** | **Used Instagram** | ***p*** | **Did not use Facebook** | **Used Facebook** | ***p*** | **Did not use more than one platform** | **Used more than one platform** | ***p*** |  |
| --- | --- | --- | --- | --- | --- | --- | --- | --- | --- | --- | --- | --- | --- |
| **Sex (% Female)** | 51.27 | 49.52 |  | 44.12 | 65.11 | *** | 40.08 | 56.04 | *** | 45.58 | 61.52 | *** |  |
| **Age (%)** |  |  | *** |  |  | *** |  |  | *** |  |  | *** |  |
| 18-29 years | 9.77 | 22.23 |  | 6.38 | 24.34 |  | 8.48 | 13.99 |  | 7.00 | 22.53 |  |  |
| 30-44 years | 22.37 | 29.58 |  | 19.57 | 32.53 |  | 24.73 | 35.61 |  | 27.34 | 41.58 |  |  |
| 45-64 years | 15.07 | 18.18 |  | 14.80 | 17.53 |  | 37.62 | 33.49 |  | 38.16 | 28.24 |  |  |
| 65 years or older | 17.81 | 14.92 |  | 19.28 | 13.02 |  | 29.18 | 16.90 |  | 27.50 | 7.64 |  |  |
| **Race/ethnicity (%)** |  |  | *** |  |  | *** |  |  | *** |  |  | *** |  |
| Asian^+^ | 5.08 | 5.97 |  | 4.47 | 6.89 |  | 6.81 | 4.53 |  | 4.82 | 6.13 |  |  |
| Black^+^ | 12.16 | 8.73 |  | 12.43 | 9.53 |  | 14.34 | 10.16 |  | 12.69 | 9.14 |  |  |
| Hispanic / Latino | 14.2 | 20.14 |  | 12.47 | 21.37 |  | 16.44 | 14.87 |  | 12.83 | 20.40 |  |  |
| White^+^ | 65.61 | 59.8 |  | 67.78 | 57.59 |  | 59.61 | 66.72 |  | 66.84 | 59.70 |  |  |
| Other groups^+^ | 2.95 | 5.36 |  | 2.85 | 4.62 |  | 2.80 | 3.73 |  | 2.82 | 4.64 |  |  |
| **Education (%)** |  |  | *** |  |  | *** |  |  | ** |  |  | *** |  |
| Less than high school | 9.34 | 4.72 |  | 9.87 | 5.43 |  | 10.19 | 7.54 |  | 10.04 | 5.13 |  |  |
| High school/ GED | 30.95 | 20.65 |  | 31.65 | 23.30 |  | 31.27 | 27.82 |  | 32.16 | 22.56 |  |  |
| Some college | 28.20 | 26.96 |  | 27.11 | 29.74 |  | 25.24 | 29.27 |  | 27.18 | 29.56 |  |  |
| Bachelor's degree or more | 31.51 | 47.67 |  | 31.37 | 41.53 |  | 33.30 | 35.37 |  | 30.61 | 42.76 |  |  |
| **Marital status (%)** |  |  | *** |  |  | *** |  |  | *** |  |  | *** |  |
| Married | 57.11 | 55.25 |  | 58.66 | 52.77 |  | 57.60 | 56.42 |  | 58.34 | 53.73 |  |  |
| Previously married^++^ | 20.78 | 13.14 |  | 21.73 | 14.22 |  | 20.15 | 18.78 |  | 21.26 | 15.20 |  |  |
| Never married | 22.11 | 31.61 |  | 19.61 | 33.01 |  | 22.25 | 24.81 |  | 20.40 | 31.07 |  |  |
| **Number of household members, *M* (*SE*)** | 1.73 (0.031) | 2.03 (0.068) | *** | 1.66 (0.033) | 2.05 (0.052) | *** | 1.57 (0.048) | 1.89 (0.034) | *** | 1.66 (0.033) | 2.03 (0.050) | *** |  |
| *Notes:* *M* = mean; *SE* = standard error; **p* < .01; ***p* < .01; ****p* < .001; GED = general equivalency diploma; ^+^non-Hispanic; ^++^separated/divorced/widowed | | | | | | | | | | | | | |
